# Study on disinfection using wet wipes and UV irradiation for areas prone to condensate accumulation in the Chinese Space Station

**DOI:** 10.3389/fmicb.2026.1850068

**Published:** 2026-06-16

**Authors:** Ying Zhang, Jiangchuan Zhou, Jinglin Ma, Lili Ren

**Affiliations:** 1 School of Life Science, Beijing Institute of Technology, Beijing, China; 2 Department of Otolaryngology Head and Neck Surgery, The 6th Medical Center of Chinese PLA General Hospital, Chinese PLA Medical School, Beijing, China

**Keywords:** China Space Station, condensate water, high-throughput microbial cultivation chip, on-orbit microbial disinfection measures, space station microbial contamination model

## Abstract

**Introduction:**

Condensate water is one of the environmental niches aboard space stations that is most prone to microbial growth. This study reports one of the first applications of a detailed characterization of the microbial composition of condensate water collected from inside the China Space Station during flight, as well as an evaluation of effective cleaning and disinfection strategies for microbial control.

**Methods:**

To maximize the recovery of microorganisms from the condensate-water environment, we developed a custom high-throughput microbial isolation and cultivation device (cChip) based on the isolation chip method (iChip) and used it for high-throughput cultivation of microorganisms from onboard areas of the China Space Station that are susceptible to microbial proliferation. In addition, to evaluate on the ground the decontamination efficacy of four disinfection methods already used in orbit (wiping with pure-water wipes, 75% alcohol wipes, quaternary ammonium salt wipes, and ultraviolet irradiation), we constructed a microbial contamination model on authentic textile flight materials using the isolated strains in proportions determined from high-throughput sequencing data. The efficacy of these four disinfection approaches against the contamination model was then assessed using contact plates, PMA-qPCR, and qPCR.

**Results:**

In total, 14 bacterial strains and 9 fungal strains were obtained. Notably, members of the genera *Oceanobacillus*, *Rhodococcus*, and *Fusarium* were not recovered by conventional isolation methods and could be isolated only with the cChip, demonstrating the strong potential of the cChip for recovering previously uncultivable microorganisms. Among the tested methods, alcohol wipes showed the highest bactericidal efficacy against bacteria in the contamination model, achieving a inactivation efficiency of 99.97%, whereas ultraviolet irradiation showed the highest fungicidal efficacy against fungi, achieving a inactivation efficiency of 99.98%.

## 1 Introduction

The space station maintains a closed environment with stable temperature and humidity, creating ideal conditions for microbial proliferation. As space station construction and operation continue to advance, microbial contamination has become an increasingly serious challenge. Microorganisms can disrupt the balance of the astronaut microbiota and induce a variety of health problems, including bacterial infections and skin allergies. In addition, they can deteriorate aerospace materials by degrading organic polymers, corroding metals, and altering the properties of electronic components, thereby reducing equipment performance or even causing system failure and substantially increasing the cost and risk of space exploration. Because of the unique space environment and the difficulty of cleaning under such conditions, uncontrolled microbial growth may threaten both astronaut health and spacecraft safety (Xin et al., 2020).

Within the unique environment of a space station, condensate water zones are among the areas most severely affected by microbial contamination. The relative humidity inside a space station is typically maintained at 40–50%, and the thermal control system contains low-temperature pipelines. When warm, humid air comes into contact with these cooled surfaces, condensate forms. Condensate collection areas provide suitable temperature and humidity and also accumulate organic matter such as astronaut metabolic byproducts. Poor ventilation and difficulty in cleaning allow condensate and dust to accumulate gradually, creating favorable conditions for microbial growth. Bacteria, fungi, and other microorganisms can proliferate rapidly in these sites and may trigger a series of serious problems. They may become airborne and threaten astronauts’ respiratory health, potentially causing pulmonary infections and related diseases, and they may also attach to equipment surfaces, corrode critical components, and interfere with normal space station operation (Novikova et al., 2006), thereby posing potential risks to space missions.

During the early design and construction of the former Soviet Mir space station, research on microbial contamination prevention and control in space habitats was still limited, which led to multiple microbial outbreak events. In one reported incident, when astronauts on Mir opened a rarely used maintenance panel with poor ventilation, they found numerous small, spherical floating water droplets inside, and these droplets were covered with dark brown microorganisms (Ott et al., 2004). Subsequent comprehensive ecological investigations of Mir demonstrated that opportunistic pathogens and environmental bacteria pathogenic to the crew were primarily derived from condensate samples rather than airborne microorganisms, with a significant proportion being Gram-negative. The major taxa identified included *Microbacterium testaceum*, *Sphingobacterium spiritivorum*, *Rhodosporidium toruloides*, *Yarrowia lipolytica*, and *Botryosphaeria ribis*. Furthermore, the microbial concentration in collected condensate samples was reported to be over 520,000-fold higher than in air samples (Kawamura et al., 2001), posing a substantial threat to both crew health and spacecraft operational safety.

To eliminate microorganisms in space station condensate water, these organisms must first be cultivated under laboratory conditions on Earth so that effective disinfection strategies can be identified. However, previous studies have shown that, for a given habitat, conventional culture media typically recover only about 1% of microorganisms, whereas approximately 99% remain uncultured (Solden et al., 2016). Several factors account for this limitation. First, the native environment is difficult to reproduce accurately; critical growth parameters, including nutrient composition and the influence of symbiotic populations, are challenging to simulate, leading to poor growth or loss of microbial viability under artificial conditions (Deng et al., 2025). Second, cultivation techniques are inherently limited: fast-growing microorganisms in conventional media rapidly consume nutrients, making it difficult for slow-growing microorganisms to form visible colonies, and microorganisms adapted to oligotrophic environments are not easily detected by standard counting or turbidimetric methods (Simu and Hagström, 2004). Third, microbial interactions are often overlooked; environmental changes can disrupt interspecies communication and symbiotic relationships, thereby restricting microbial growth (Mark Welch et al., 2016). Fourth, cultivation time may be insufficient, as the recovery efficiency of some microorganisms is positively associated with incubation duration, and prolonged cultivation can improve recovery (El-Naggar et al., 2015). If microorganisms cannot be isolated from condensate water, the efficacy of existing disinfection approaches cannot be properly evaluated, nor can new, targeted, and highly effective control strategies be developed on the basis of microbial characteristics.

With advances in molecular biology, culture-independent methods for analyzing environmental microbial communities have revealed a much broader diversity of previously uncultivable microorganisms; however, these methods cannot provide pure cultures. The development of the isolation chip (iChip) method opened a new route for isolating and cultivating previously uncultivable microorganisms. The high-throughput iChip device was developed by D. Nichols in 2010 and was specifically designed for the isolation of so-called “uncultivable” microorganisms (Nichols et al., 2010). Our research group subsequently modified the iChip and developed the cChip, making it more suitable for isolating and cultivating microorganisms from condensate water samples. The cChip provides structural support sites and growth space for microorganisms, facilitates enrichment cultivation in aquatic environments, and avoids the conventional serial-dilution preprocessing steps that can prevent low-abundance bacteria from being recovered (Zhao et al., 2023; Lodhi et al., 2023).

In this study, condensate water samples collected during the operational phase of the space station were used as the research material. To our knowledge, the cChip was one of the first applied for high-throughput cultivation and identification of microbial isolates from these in-flight condensate samples. In parallel, individually cultured isolates were combined to simulate the community structure of microbial contamination on cabin surfaces, and a surface microbial contamination model with a microbial load of 4 × 10^9^ CFU/100 cm^2^ was constructed on authentic space-station textile materials. Four disinfection methods currently used on the space station—ultraviolet irradiation, pure-water wipes, 75% alcohol wipes, and quaternary ammonium salt wipes—were then evaluated against this contamination model. Residual microorganisms were assessed by microbial culture and qPCR, thereby verifying the decontamination efficacy of the different disinfection methods.

## 2 Materials and methods

### 2.1 Sampling site and procedure

**Figure 1:**
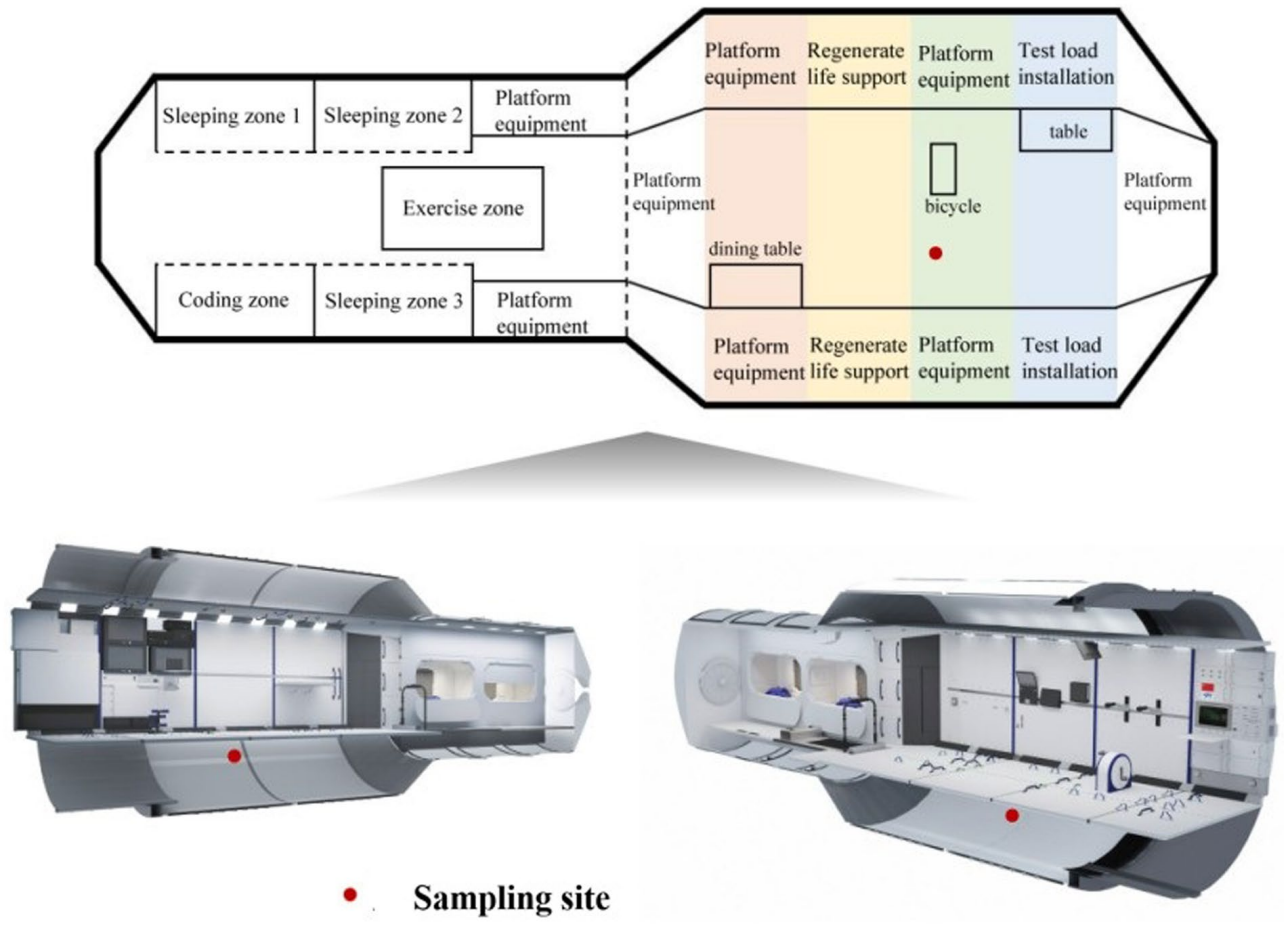
Schematic diagram of the sampling site. The two panels show 2D and 3D structural schematics of the space station module, illustrating the distribution of the large-diameter section, the small-diameter section, and the internal instrument facilities within the core module. The red dot indicates the sampling site, which was located in the condensate accumulation area beneath the floor of the large-diameter section of the core module.

Based on the actual dimensions of the China Space Station, a 3D structural model of the interior was constructed using 3ds Max software to map the distribution of the large-diameter section, small-diameter section, and internal instrument facilities within the core module (Figure 1) (Zhang et al., 2025). The core module was selected as the target area for condensate collection. Condensate samples were collected by swabbing the exterior of the low-temperature condensate piping with sterile cotton strips. The collected samples were sealed in sterile sampling bags, transported back to Earth aboard the crew return capsule 1 week later, and transferred to the laboratory within 24 h. All downstream microbial isolation and processing were completed within 12 h of sample receipt.

### 2.2 Design of the cChip

**Figure 2:**
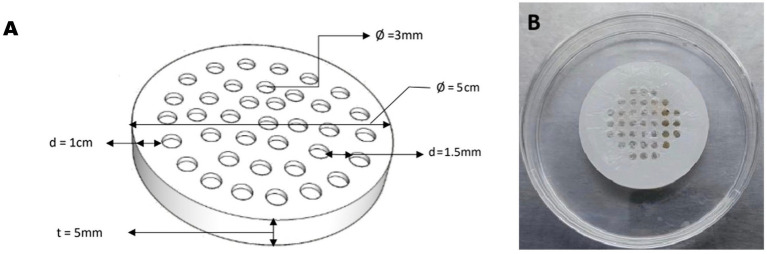
Structural design **(A)** and photograph **(B)** of the cChip. The cChip can simulate the natural environment for microbial growth under laboratory conditions and is suitable for cultivating microorganisms present in condensate water samples.

The high-throughput cultivation chip (cChip) utilized in this study was designed using Adobe Illustrator CC 2015 (version 19.0.0) (Figure 2). To optimize the cChip for the cultivation of condensate-derived microorganisms, several structural parameters were refined based on previous iterations (Zhao et al., 2023). The optimized cChip was fabricated from polypropylene plastic with a diameter of 5 cm, containing multiple cultivation wells (3 mm in diameter and 5 mm in depth). The upper and lower clamping plates originally used to secure the membrane were eliminated. Instead, the bottom of the cChip was directly sealed with a 0.03 μm pore-size polycarbonate track-etched (PCTE) membrane (Whatman, Catalog No. 10417006) using RTV 108 silicone adhesive.

### 2.3 Isolation, purification, and identification

**Figure 3:**
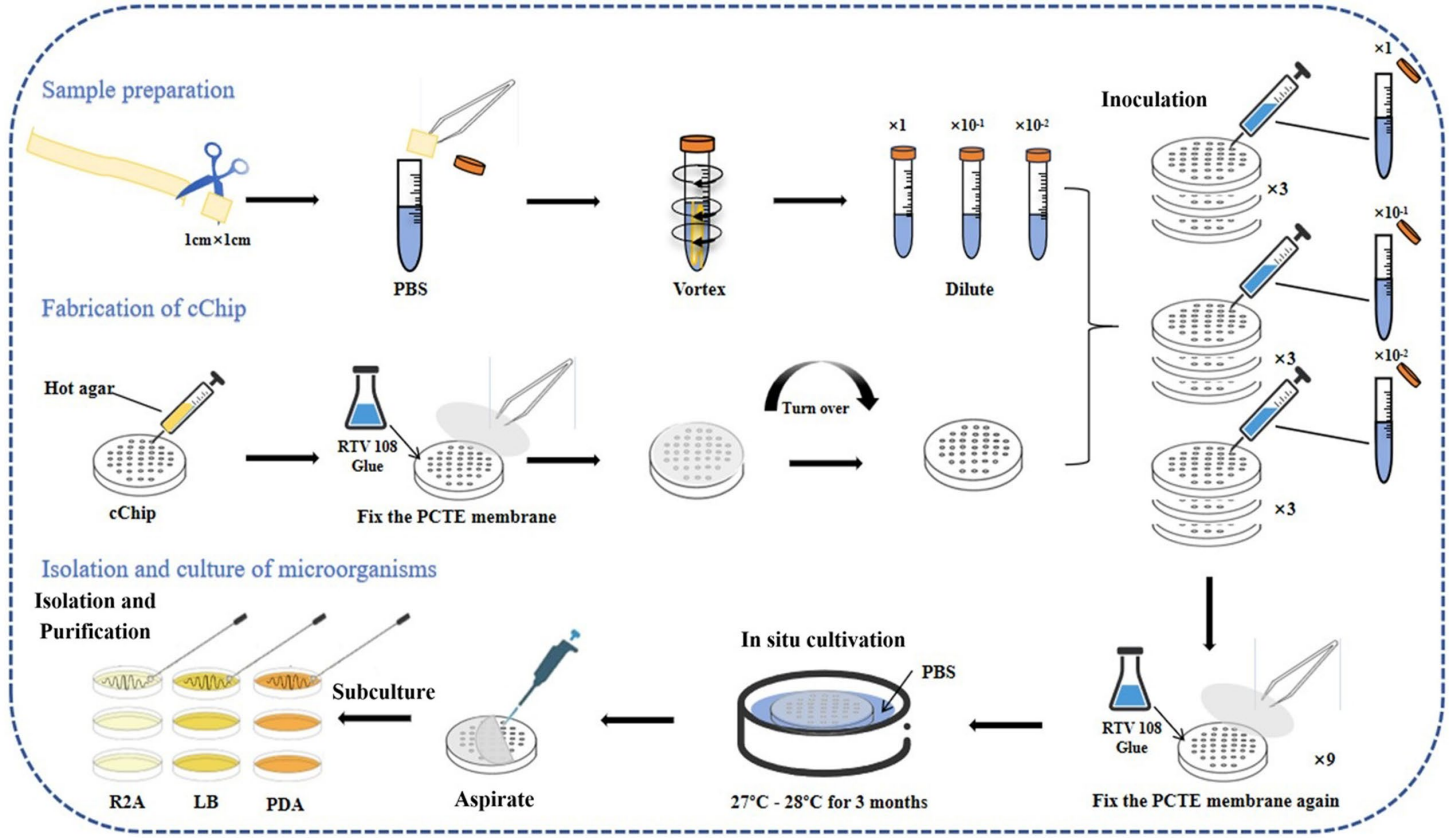
Workflow for microbial cultivation using the cChip.

As shown in Figure 3, a 1 cm × 1 cm section of the sampled cotton strip was aseptically excised using sterile surgical scissors and immersed in 5 mL of sterile PBS. The suspension was vortexed for 15 min to thoroughly elute the microbial biomass. The resulting eluate was serially diluted to prepare three concentrations: the original eluate, 10^−1^, and 10^−2^.

Aliquots of molten agar were dispensed into each cultivation chamber of the cChip using a syringe. After the agar solidified, the bottom was sealed with the PCTE membrane. The cChip was inverted, and 10 μL of each sample dilution was inoculated into the respective cultivation chambers and agitated to ensure homogenous mixing. Three technical replicates (parallel chips) were prepared for each dilution. The top surface of the cChip was subsequently sealed with a PCTE membrane. The assembled cChips were submerged in sterile PBS and incubated at 27–28 °C for 3 months. The surrounding PBS was replaced biweekly to maintain an oligotrophic environment and sustain microbial viability.

Following incubation, the top membrane was removed, and the microbial growth from each chamber was streaked onto LB, R2A, and PDA plates using sterile pipette tips. Plates were incubated at 37 °C (LB) and 28 °C (R2A and PDA), and colony growth was monitored every 12 h. Distinct single colonies were isolated, purified by repeated streaking, and preserved.

For bacterial identification, genomic DNA was extracted using the DP302 Bacterial DNA Kit (Tiangen, China), and the 16S rRNA gene was amplified using universal primers P0 and P6. For fungal isolates, mycelia were cultivated in SDA liquid medium (28 °C, 12–48 h), harvested by centrifugation (12,000 rpm, 2 min), snap-frozen in liquid nitrogen for 5 min, and homogenized using a bead mill with sterile steel beads for 5 min. Fungal genomic DNA was extracted using the DP320-02 Plant Genomic DNA Kit (Tiangen, China) and amplified using universal ITS primers (ITS1/ITS4). Sterile water was used as the negative template control for all PCR assays. Amplicons were subjected to Sanger sequencing (BGI, Liuhe, China), and the resulting sequences were queried against the NCBI GenBank database using BLAST. Phylogenetic trees were constructed using MEGA11. Initial trees were inferred using the Maximum Likelihood and Neighbor-Joining methods, and the final phylogenetic tree was calculated based on the General Time Reversible model (Nei and Kumar, 2000). Bootstrap analysis was performed with 1,000 replicates, and the support threshold was set at 50%.

### 2.4 Preparation of the microbial contamination model for cabin interior surfaces

Because microorganism-carrying condensate water can float within the cabin and contaminate interior surfaces, we constructed a microbial contamination model on Metasbu fabric aerospace material. This model was established based on the high-throughput 16S rRNA gene amplicon sequencing results of a space station surface sample obtained in our previous study (Zhang et al., 2025), as well as empirical data from our previous metagenomic analyses of microorganisms in the space environment. Specifically, bacteria from the four genera accounting for the top 99.9% of the total bacterial abundance and fungi accounting for 0.1% of the microbial community were selected.

The contamination model was prepared as follows. Four bacterial strains isolated using the cChip method were selected, as shown in Table 1. Standard curves between OD_600_ values and bacterial concentrations were generated for these strains to enable subsequent proportional preparation of the microbial suspension. In addition, nine fungal strains belonging to four genera and isolated using the cChip method were selected, as shown in Table 1. After liquid cultivation, their spore suspensions were quantified for subsequent proportional preparation of the microbial suspension.

**Table 1:** Relative abundance of microorganisms included in the contamination model.

Selected strains	Proportion in the total biomass
*Paenibacillus* sp. SB45-2B	52.1%
*Pantoea agglomerans* strain BQC01	42.5%
*Bacillus velezensis* strain XC1	3.5%
*Rhodococcus corynebacterioides* strain 045	1.8%
*Alternaria alternata* isolate TY172-17	0.011%
*Alternaria* sp. isolate ZY01	0.011%
*Chaetomium globosum* isolate CES5	0.011%
*Fusarium oxysporum* strain EP19	0.011%
*Fusarium* sp. isolate CDCF2523	0.011%
*Fusarium* sp. strain p14	0.011%
*Fusarium verticillioides* isolate G4	0.011%
*Fusarium verticillioides* isolate PFSRFv102	0.011%
*Punctularia subhepatica* isolate T6	0.011%

The proportions of the bacterial strains in the contamination model were determined according to the high-throughput 16S rRNA gene amplicon sequencing results. Because fungi represented only 0.1% of the total microbial abundance, the nine fungal strains were mixed into the contamination model at equal proportions. The final volume of the suspension was 1 mL, and the total concentration of microorganisms was 1 × 10^9^ cells/mL. The final concentrations of *Paenibacillus*, *Pantoea*, *Bacillus*, and *Rhodococcus* were 5.21 × 10^8^cells/mL, 4.25 × 10^8^cells/mL, 3.5 × 10^7^cells/mL, and 1.8 × 10^7^cells/mL, respectively. Each of the nine fungal strains reached a final concentration of 1.1 × 10^5^ cells/mL in the contamination suspension.

Nomex aerospace-grade textile, identical to the interior fabrics used in the China Space Station, was utilized as the contamination substrate. The Nomex fabric was aseptically cut into 5 cm × 5 cm squares, wrapped in aluminum foil, and sterilized by autoclaving (121 °C, 30 min). Within a Class II biosafety cabinet, the microbial suspension was uniformly applied to the Nomex squares using a sterile atomizer. The inoculated textiles were air-dried in sterile Petri dishes to establish a final contamination load of 4 × 10^9^cells/100 cm^2^.

### 2.5 Disinfection methods

The decontamination efficacy of four standard space station disinfection protocols was evaluated: sterile purified water wipes (Qingfeng™), 75% alcohol disinfecting wipes (Deyou™), sterile quaternary ammonium surface wipes (Little Touch™), and ultraviolet (UV) irradiation (Table 2).

**Table 2:** Disinfection methods and active ingredients.

Disinfection method	Disinfection material	Active ingredient(s)
Pure-water wipe disinfection	Qingfeng™ EDI Pure Water Wipes	Purified water
Alcohol wipe disinfection	Deyou™ 75% Alcohol Disinfecting Wipes	75% alcohol
Ultraviolet disinfection	30 W ultraviolet low-pressure mercury germicidal lamp	Ultraviolet radiation
Quaternary ammonium wipe disinfection	Little Touch™ sterile surface wipes	Purified water, organosilicon quaternary ammonium salt (0.05%), benzalkonium chloride (0.13%), glycerol

#### 2.5.1 Wipe-based disinfection

The contaminated aerospace textiles were systematically wiped in three directions (horizontal, vertical, and diagonal). The operator applied a consistent mechanical pressure (approximately 4.9 N) to ensure maximal contact between the functional side of the wipe and the fabric, taking care to avoid cross-contamination from the gloves or the reverse side of the wipe. The treated fabrics were air-dried at room temperature.

#### 2.5.2 UV disinfection

Contaminated samples were exposed to a 30 W low-pressure mercury germicidal lamp at a distance of 40 cm for 20 min inside a biosafety cabinet (with the glass sash closed and internal illumination disabled).

## 3 Results

### 3.1 Identification of culturable microorganisms from a spaceflight microbial hotspot

**Figure 4:**
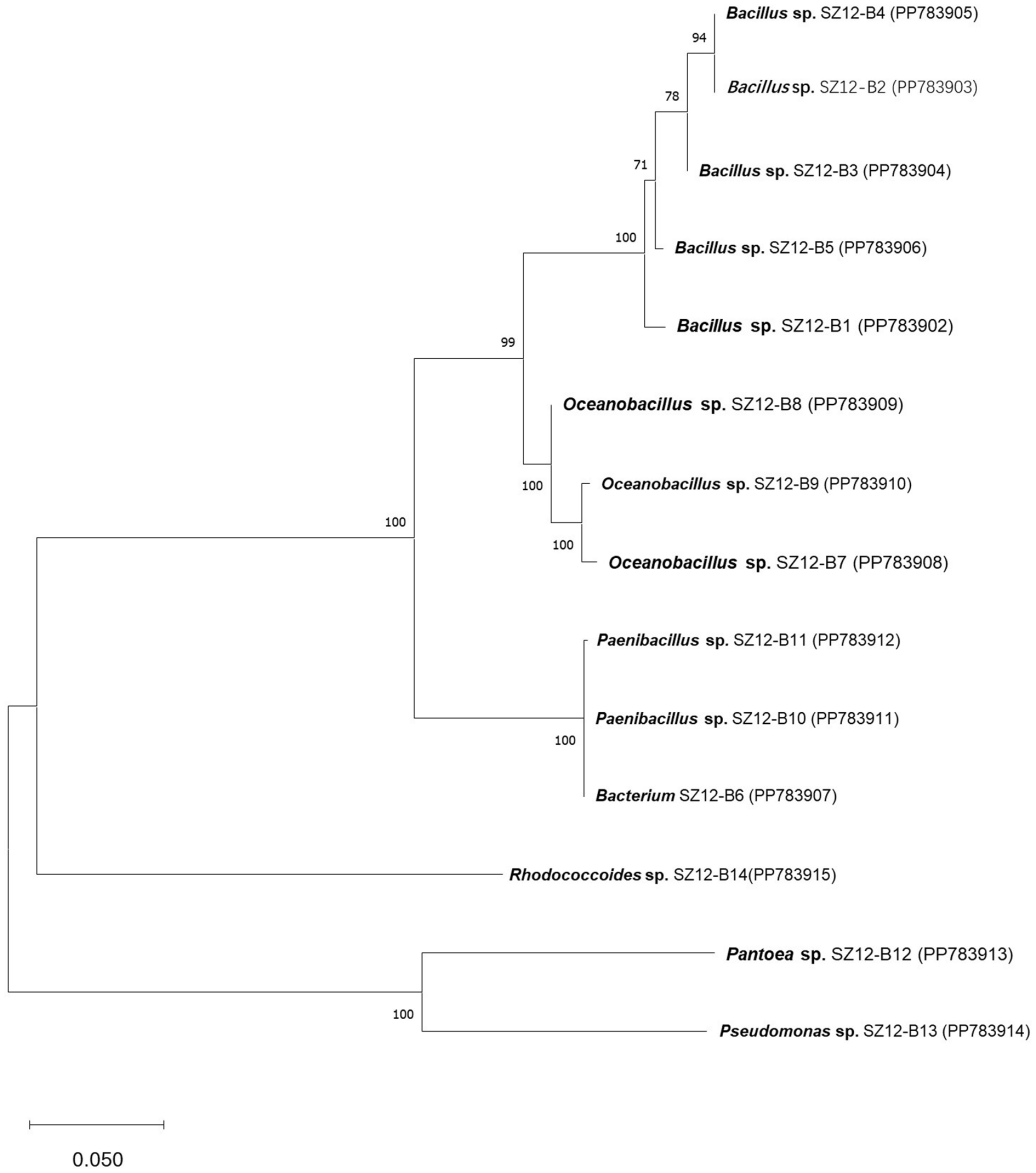
Phylogenetic tree of the 14 bacterial strains identified from microbial samples collected from the condensate region of the space station using the cChip cultivation method. The tree was generated using MEGA11 based on the general time reversible model. Bootstrap analysis was performed with 1,000 replicates, with the bootstrap threshold set at 50%.

**Figure 5:**
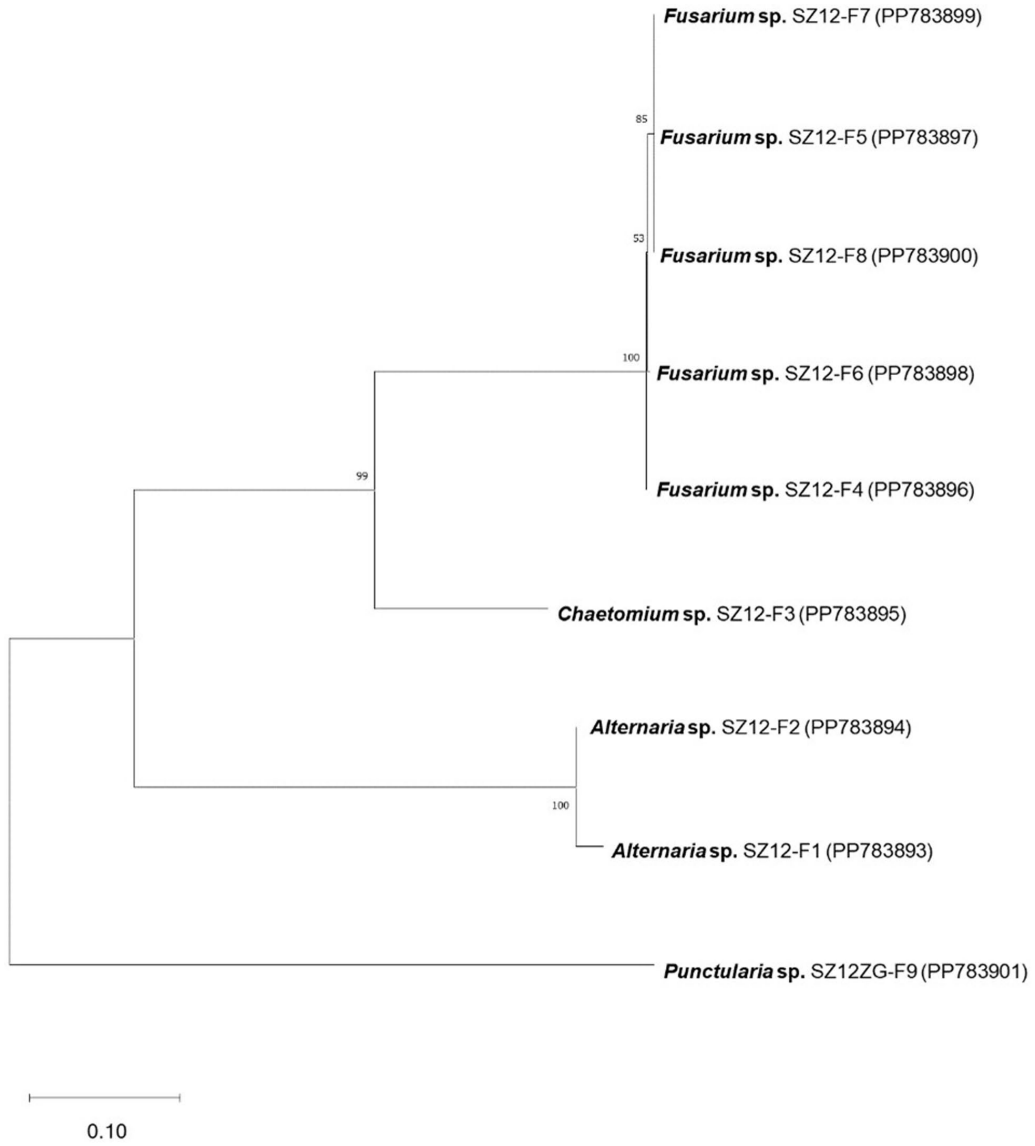
Phylogenetic tree of the nine fungal strains identified from microbial samples collected from the condensate region of the space station using the cChip cultivation method. The tree was generated using MEGA11 based on the general time reversible model. Bootstrap analysis was performed with 1,000 replicates, with the bootstrap threshold set at 50%.

Microbial samples collected from the condensate region of the space station were cultured using both the conventional spread plating method and the cChip method. For bacterial and fungal isolates, the 16S rRNA gene and ITS region, respectively, were amplified. After sequencing, the resulting amplicons were compared with sequence information in the GenBank database to obtain the basic taxonomic information for each isolate, and phylogenetic trees were constructed (Figure 4). Using the cChip cultivation method, 14 bacterial strains were identified, including five strains belonging to the genus *Bacillus*, one to *Rhodococcus*, one to *Pseudomonas*, one to *Pantoea*, three to *Oceanobacillus*, and two to *Paenibacillus*. The cChip method also resolved the phylogenetic positions of nine fungal strains (Figure 5), including two strains of *Alternaria*, one of *Chaetomium*, two of *Punctularia*, and four of *Fusarium*. By contrast, the conventional spread plating recovered only eight bacterial strains and four fungal strains from the same sample. All microorganisms obtained by the conventional spread plating were also recovered by the cChip. Notably, *Oceanobacillus*, *Rhodococcus*, and *Fusarium* were not isolated by the conventional method and could be recovered only by the cChip.

### 3.2 Culture-based analysis of residual microorganisms after disinfection

A microbial contamination model was established on a 5 cm × 5 cm piece of flight material by uniformly spraying the microbial suspension onto its surface; the detailed procedure is described in the Materials and Methods section. The contamination level was set at 4 × 10^9^ CFU/100 cm^2^, which was the lowest microbial contamination level macroscopically visible. A positive control group was established by spraying the contamination suspension without subsequent disinfection, and the bactericidal/fungicidal effects of four disinfection methods were compared experimentally.

**Figure 6:**
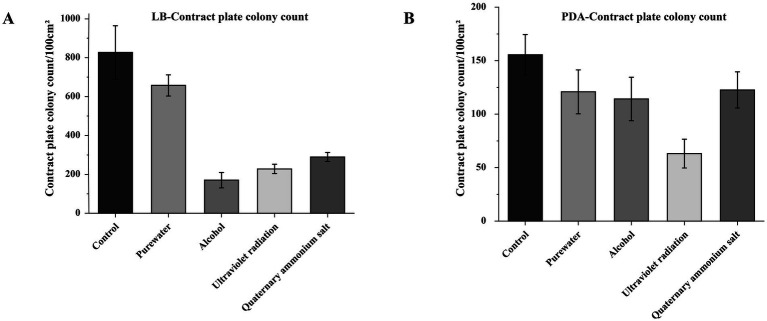
Colony counts of bacteria **(A)** and fungi **(B)** recovered using contact plates. LB and PDA contact plates were used to enumerate bacterial and fungal colonies, respectively. From left to right, the different shades of gray represent the untreated control group, the pure-water wipe control group, the alcohol wipe disinfection group, the ultraviolet disinfection group, and the quaternary ammonium wipe disinfection group. The *y*-axis indicates the number of colonies recovered from each 100 cm^2^ surface area by the contact plates.

After treatment with pure-water wipes, 75% alcohol wipes, ultraviolet irradiation, and quaternary ammonium salt wipes, the residual bacterial concentrations collected using LB contact plates were 657.32 (±154.32) CFU/100cm^2^, 170.24 (±111.68) CFU/100cm^2^, 228.00 (±67.08) CFU/100cm^2^, and 289.32 (±65.00) CFU/100cm^2^, respectively. The bacterial concentration in the positive control group was 828.68 (±39.09) CFU/100cm^2^. The residual fungal concentrations collected using PDA contact plates after the same four treatments were 120.88 (±58.00) CFU/100cm^2^, 114.24 (±57.44) CFU/100cm^2^, 63.12 (±38.16) CFU/100cm^2^, and 122.68 (±47.92) CFU/100cm^2^. The fungal concentration in the positive control group was 155.56 (±52.24) CFU/100cm^2^ (Figure 6; Supplementary Table S1).

Meanwhile, after treatment with pure-water wipes, 75% alcohol wipes, ultraviolet irradiation, and quaternary ammonium salt wipes, the residual bacterial concentrations detected by qPCR were 1.27 (±0.39) × 10^8^ copies /100 cm^2^, 2.96 (±0.46) × 10^7^ copies /100 cm^2^, 4.20 (±0.36) × 10^7^ copies /100 cm^2^, and 6.00 (±0.68) × 10^7^ copies /100 cm^2^, respectively. The bacterial concentration in the positive control group was 2.92 (±0.39) × 10^8^ copies /100 cm^2^.

The residual fungal concentrations detected by qPCR were 2.37 (±0.23) × 10^6^ copies /100 cm^2^, 3.03 (±0.48) × 10^6^ copies /100 cm^2^, 6.80 (±0.32) × 10^6^ copies /100 cm^2^, and 3.19 (±0.57) × 10^6^ copies /100 cm^2^, respectively. The fungal concentration in the positive control group was 7.96 (±0.28) × 10^6^ copies /100 cm^2^.

The residual concentrations of viable microorganisms were further detected using PMA-qPCR. After treatment with pure-water wipes, 75% alcohol wipes, ultraviolet irradiation, and quaternary ammonium salt wipes, the residual viable bacterial concentrations on the aerospace material surfaces were 9.29 (±1.42) × 10^5^ copies /100 cm^2^, 8.25 (±0.57) × 10^3^ copies /100 cm^2^, 7.21 (±1.57) × 10^4^ copies /100 cm^2^, and 1.50 (±0.13) × 10^5^ copies /100 cm^2^, respectively. The viable bacterial concentration in the positive control group was 1.33 (±0.04) × 10^6^ copies /100 cm^2^.

**Figure 7:**
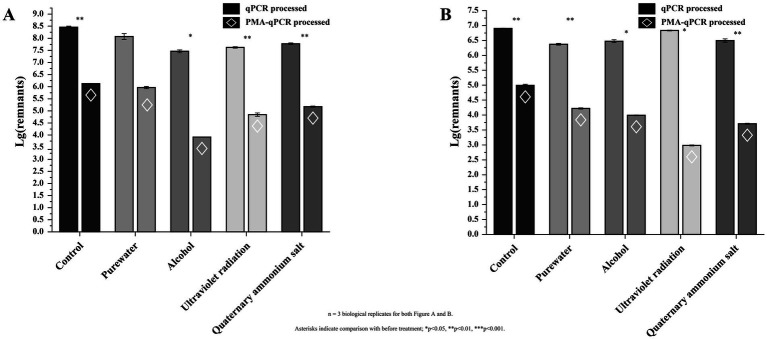
Copy numbers of bacterial 16S rRNA genes and fungal ITS genes after disinfection, as determined by qPCR and PMA-qPCR. **(A)** Comparison of total bacterial copy numbers (left) and viable bacterial copy numbers (right) on the surface after disinfection treatment. **(B)** Comparison of total fungal copy numbers (left) and viable fungal copy numbers (right) on the surface after disinfection treatment. From left to right, the different colors in the figure represent the untreated control group, pure-water wipe control group, alcohol wipe disinfection group, ultraviolet disinfection group, and quaternary ammonium salt wipe disinfection group, respectively. Within each group, the left bar indicates the total copy number detected by qPCR, and the right bar indicates the viable copy number detected by PMA-qPCR. The y-axis represents the log_10_-transformed copy number values.

The residual viable fungal concentrations were 1.67 (±0.11) × 10^4^ copies /100 cm^2^, 9.82 (±0.44) × 10^3^ copies /100 cm^2^, 9.62 (±0.56) × 10^2^ copies /100 cm^2^, and 5.10 (±0.29) × 10^3^ copies /100 cm^2^, respectively. The viable fungal concentration in the positive control group was 9.91 (±1.24) × 10^4^ copies /100 cm^2^ (Figure 7; Supplementary Table S2).

## 4 Discussion

### 4.1 Potential of the cChip for microbial isolation

The cChip operates by simulating the native environment for microbial growth, thereby allowing previously uncultivable microorganisms to establish chemical communication with their surrounding environment under these simulated conditions. As a modified version of the iChip, the cChip was redesigned by removing the upper and lower plates originally used to secure the PCTE membrane and instead directly sealing the membrane to the chip surface with RTV 108 adhesive, while also increasing the diameter of the cultivation wells. The purpose of these modifications was to improve material exchange between the inside and outside of each cultivation well as much as possible while still preventing direct exchange of microorganisms between the well interior and the external environment (Zhao et al., 2023). Compared with conventional methods, the cChip has the advantage of enabling cultivation under conditions that better mimic the original habitat. Because this microbial community was obtained from the condensate region, PBS buffer was used during cultivation to prevent certain microorganisms from lysing in pure water while at the same time reproducing, as closely as possible, the nutrient-poor conditions of condensate water. In this way, the cultivation process functioned as a form of simulated *in situ* cultivation, allowing the experimental results to better reflect the growth characteristics of microorganisms in the condensate environment.

In addition, the small but numerous cultivation wells facilitate the partitioning of microorganisms into a large number of independent microenvironments, which is favorable for the growth of individual colonies. Each cultivation well in the cChip represents an independent cultivation unit. Although material exchange can occur between the well and the external environment, microorganisms growing within a given well cannot leak into the surrounding environment through the PCTE membrane, nor can microorganisms be exchanged between wells. As the dilution increases, the microbial concentration within each well gradually decreases, and interspecies competition is correspondingly reduced. This enables some microorganisms that would otherwise fail to grow under conventional culture conditions because of competition with other species to grow successfully, thereby allowing the isolation of microorganisms that are effectively uncultivable by traditional methods. Because the chip contains a large number of cultivation wells, the number of microorganisms that can potentially be isolated from a single chip is also markedly greater than that obtained by conventional culture methods, thus improving isolation efficiency.

Based on the findings of this study, we conclude that the cChip has strong potential for microbial isolation, particularly for microorganisms that cannot be recovered using conventional methods. Compared with the traditional plate isolation method, the cChip enabled the recovery of six additional bacterial strains and five additional fungal strains, including members of *Oceanobacillus*, *Rhodococcus*, and *Fusarium*, which were not detected by conventional cultivation. These results clearly demonstrate the potential of the cChip for isolating microorganisms that are not recoverable by traditional methods.

### 4.2 Environmental and human health impacts of microorganisms in condensate water

This study found that the condensate-associated microbial community included organisms with potential health risks to humans and potential corrosive effects on the surrounding environment. Among the bacteria, some species within the genus *Rhodococcus* are capable of infecting humans and causing respiratory tract infections (Lin et al., 2019); some species within the genus *Pseudomonas* may cause suppurative lesions, bacteremia, and multi-organ disease (Ramesh et al., 2025); the isolated strain *Pantoea agglomerans* is a pathogen of certain crops and is also pathogenic to humans (Cruz et al., 2007); and some species within the genus *Paenibacillus* may have the capacity to degrade cellulose and corrode aerospace materials (Shakir et al., 2022). Among the fungi, the isolated strains *Fusarium oxysporum* and *Fusarium verticillioides* are plant pathogens that can cause wilting diseases in a variety of crops (Aoki et al., 2014); the isolated strain *Alternaria alternata* is also a plant pathogen and can produce toxins such as tenuazonic acid (TeA), thereby contaminating food products (Partsch et al., 2025); and the isolated strain *Chaetomium globosum* is a common indoor contaminating fungus that may induce allergic reactions, infect superficial human tissues, and produce cellulases (Aoki et al., 2014), thereby potentially contributing to the deterioration of certain cabin materials. Taken together, these findings indicate that microorganisms present in condensate water warrant close attention.

### 4.3 Significance of the disinfection experiments under real-world conditions

Using the cChip, we recovered as many microorganisms from condensate water as possible. To reproduce, as closely as possible, the original community structure of microbial contamination formed when condensate-associated microorganisms contaminate space station interior surfaces, we had previously characterized the microbial community structure of interior surface samples from the China Space Station by high-throughput 16S rRNA amplicon sequencing. Based on community composition data from selected areas, we then artificially constructed a contamination model using microorganisms isolated from condensate water, such that the relative abundance of each microorganism in the simulated contamination suspension approximated that of the original surface community as closely as possible.

Conventional approaches generally rely on cultivation, enrichment, and serial passaging of the original microbial community, after which the expanded community suspension is used in subsequent contamination and disinfection studies. Compared with our contamination-modeling strategy, however, conventional methods have two major limitations. First, during enrichment of the microbial community, it is difficult to maintain conditions that are fully consistent with the original environment. When conventional culture media are used, changes in growth conditions may alter the dominant taxa and thereby shift community structure during cultivation. Conversely, if the original community environment is used—namely, a nutrient-poor condensate-like environment—nutrient limitation may lead to failed enrichment, preventing the community from reaching the desired concentration. Second, because this community contains difficult-to-culture microorganisms, conventional enrichment procedures are likely to hinder the growth of these organisms, again resulting in changes in community composition. In contrast, the contamination model established in this study was based on single-strain cultivation using the cChip together with original community-structure data. This strategy allowed us to preserve the community structure as much as possible while also taking advantage of the cChip to better retain the representation of microorganisms that are difficult or impossible to culture by conventional methods.

According to the cChip-based isolation and cultivation results obtained in this study, one bacterial strain belonging to the genus *Rhodococcus* and five fungal strains belonging to the genus *Fusarium* included in the contamination model could only be isolated using the cChip method, whereas microorganisms from these two genera were not recovered by conventional cultivation methods. Although difficult-to-culture microorganisms are difficult to isolate using conventional methods, they objectively exist within the microbial community responsible for contamination. Moreover, microorganisms belonging to different species or genera may exhibit different levels of tolerance to disinfection. Therefore, incorporating these difficult-to-culture microorganisms into the contamination model can more realistically reflect the disinfection efficacy against actual contamination caused by condensate water. Taken together, the approach of isolating as many microorganisms as possible and constructing a contamination model to simulate real contamination conditions may be more feasible for this study.

In this study, we uniformly sprayed a simulated contamination suspension at a concentration of 10^9^CFU/mL onto 25cm^2^ fabric pieces. The significance of this concentration is that when a microbial colony contains approximately 10^9^CFU, it becomes macroscopically visible to the crew and therefore constitutes an onboard contamination event that is likely to be noticed and to warrant disinfection. To ensure accuracy, residual microorganisms after disinfection were quantified using both the contact plate method and qPCR/PMA-qPCR. Contact plates are widely used in microbiology for surface microbial enumeration and are also used on the International Space Station.

Because the microbial concentration in eluates recovered from the flight materials was too low to yield colonies within the countable range on culture media (30–300 colonies per plate) (Da Silva et al., 2018), the conventional spread-plate method commonly used in laboratories for microbial enumeration was not suitable. Under these conditions, however, the contact plate method can effectively recover and enumerate culturable microorganisms. Therefore, when the residual microbial concentration is unknown, the contact plate method may be more advantageous than the traditional spread-plate method for maximizing the recovery and enumeration of culturable microorganisms.

According to a previous study (Xin et al., 2020), when the surface microbial concentration is low, contact plate counting may provide more reliable results than molecular detection methods. However, when the microbial concentration is high, the number of distinguishable colonies on the contact plate is limited by its surface area. In addition, microbial competition during growth on the contact plate may further affect colony formation. When colonies grow too close to one another, some faster-growing colonies may occupy the growth space of slower-growing microorganisms and consume nutrients in the surrounding environment, causing some colonies to grow poorly or slowly. As a result, these colonies may fail to reach a visually detectable size and therefore may not be counted (Berdy et al., 2017; Chaudhary et al., 2019). Consequently, contact plate counting may underestimate the actual microbial load under high-concentration conditions.

qPCR and PMA-qPCR are likewise commonly used methods for microbial quantification, with qPCR being used to determine total microbial load and PMA-qPCR being used primarily to quantify viable microorganisms.

By simultaneously monitoring qPCR and PMA-qPCR data, the proportion of viable microorganisms relative to the total microbial load after disinfection can be determined under largely consistent detection conditions, thereby allowing the disinfection efficiency to be calculated. When the microbial concentration is too low, molecular detection methods may fail to detect microbial abundance because of insufficient genomic DNA extraction efficiency. Therefore, molecular detection results are more accurate and reliable only when the microbial concentration is relatively high.

Contact plate culture is well suited for monitoring low-concentration microbial contamination, but it is not suitable for detecting high-concentration microbial contamination. The upper detection limit of the contact plate method is on the order of 10^3^, whereas the lower detection limit of qPCR is approximately 10^2^ (Xin et al., 2020). Taken together, contact plate culture and qPCR-based molecular detection are, respectively, applicable to lower and higher microbial concentrations, and the two methods can effectively complement each other.

In this study, the microbial loads detected by qPCR and PMA-qPCR were markedly higher than those obtained by contact plate culture, indicating that the residual microbial concentration on the surface after one round of disinfection remained relatively high, reaching the order of 10^5^. This exceeded the applicable upper detection limit of contact plate culture, which is approximately 10^3^, thereby leading to an underestimation by the culture-based method. Therefore, the PMA-qPCR data in this study more closely reflected the actual microbial load.

Based on the results of residual microbial detection after disinfection, we found that alcohol wipes were the most effective method for bacterial decontamination, whereas ultraviolet irradiation was the most effective for fungal decontamination. Alcohol exerts its antimicrobial effect by allowing ethanol molecules to diffuse freely across the cell membrane, causing dehydration and denaturation of intracellular and membrane-associated proteins (Boyce, 2018). Ultraviolet disinfection, in contrast, acts primarily through direct damage to intracellular genetic material. Because bacteria are structurally simpler prokaryotic unicellular organisms, they are generally more susceptible to alcohol-induced damage and inactivation than fungi, which have more complex cellular structures (Thomas, 2012). In addition, fungal spores are typically highly dehydrated and possess thick cell walls, making it difficult for alcohol to penetrate into the spores and exert an effective inactivation effect. Once environmental conditions improve, the spores can germinate and fungal growth can resume. Differences in cell wall composition between bacteria and fungi may also explain why fungi are generally more resistant than bacteria to various disinfectants. Bacterial cell walls are composed mainly of peptidoglycan, whereas fungal cell walls are composed primarily of chitin; compared with peptidoglycan, chitin provides stronger protection for fungal cells (Wan et al., 2022; Dai et al., 2015). Ultraviolet irradiation, by directly targeting genetic material, may be particularly effective against fungi because fungi are eukaryotes with more complex genetic organization and weaker self-repair capacity. By contrast, bacteria, as prokaryotes, possess mechanisms such as the SOS response and photoreactivation pathways (Li et al., 2017), which can partially repair damage to genetic material and thereby improve survival.

### 4.4 Limitations in the design of the contamination model

#### 4.4.1 Proportional design of the contamination model

During the construction of the contamination model, our previous metagenomic sequencing analyses of microorganisms in the space environment showed that fungi generally accounted for approximately 0.1% of the microbial community in space station surface samples. Therefore, in the contamination model, the proportions of bacteria and fungi were set at 99.9 and 0.1%, respectively.

For the proportional design of different bacterial taxa in the contamination model, the relative abundances of each genus were determined based on the high-throughput 16S rRNA gene amplicon sequencing results of space station surface samples obtained in our previous work. At present, there may be no optimal method for accurately converting 16S rRNA gene copy number abundance in a microbial community into CFU values. Therefore, in this study, the bacterial composition of the contamination model was directly established according to the relative abundance of 16S rRNA genes.

For the proportional design of different fungal taxa in the contamination model, because fungi accounted for only 0.1% of microorganisms in the space environment, the model was simplified to some extent. Specifically, the nine isolated fungal strains were directly mixed into the contamination model at equal proportions.

#### 4.4.2 Disinfection methods and efficacy evaluation

During the evaluation of disinfection efficacy, the following procedure was adopted. A simulated contamination suspension with a defined microbial concentration was prepared according to the designed proportions and then sprayed onto the surface of the aerospace material. The contaminated material surface was subsequently subjected directly to disinfection treatment, after which the residual microbial load was determined using contact plate culture, qPCR, and PMA-qPCR.

In this study, microorganisms were not allowed to grow on the aerospace material surface before disinfection. This was because the proportions of different microorganisms in the contamination model needed to be controlled to approximate the actual contamination scenario as closely as possible. If microorganisms were allowed to grow on the aerospace material surface, the relative abundance of different microorganisms might change, and the total microbial load would also become difficult to control. Therefore, this study did not simulate the long-term growth of microorganisms on space station material surfaces.

During microbial community growth under the microgravity conditions of the space environment, various changes may occur, including biofilm formation, changes in growth rates, and shifts in dominant populations (Chen et al., 2024; Shakir et al., 2022). Nevertheless, considering that different microorganisms may exhibit different levels of tolerance to different disinfection methods, the contamination model in this study was constructed primarily to ensure controlled microbial species composition and microbial quantity. In future studies, long-term cultivation under simulated microgravity conditions could be considered, with monitoring of the total microbial load, the relative abundance of different microorganisms, and biofilm formation. Existing disinfection methods could then be applied, followed by detection to evaluate changes before and after disinfection.

With respect to disinfection methods, commonly used approaches for surface microbial contamination in humid or aqueous environments generally include chlorine, chloramine, ozone, peroxides, and ultraviolet disinfection (Isabel et al., 2024). However, because only ultraviolet irradiation and wipe-based disinfection are currently permitted for surface disinfection in the on-orbit environment, this study evaluated only these two categories of disinfection methods in order to identify the most suitable strategy.

## 5 Conclusion

In this study, a novel high-throughput *in situ* cultivation-based microbial chip (cChip) was used to achieve high-throughput cultivation and identification of microorganisms from condensate water collected aboard the China Space Station. A total of 10 genera were recovered: *Bacillus*, *Rhodococcus*, *Pseudomonas*, *Pantoea*, *Oceanobacillus*, *Paenibacillus*, *Alternaria*, *Chaetomium*, *Punctularia*, and *Fusarium*. The cChip showed strong potential for the isolation of previously uncultivable microorganisms. In addition, using the cultivated isolates, we established a surface microbial contamination model on Nomex flight textile material and evaluated the decontamination efficacy of four currently used disinfection methods: ultraviolet irradiation, pure-water wipes, 75% alcohol wipes, and quaternary ammonium wipes. Using culture-based methods together with qPCR and PMA-qPCR to quantify total residual microorganisms and viable residual microorganisms after disinfection, we found that 75% alcohol wipes showed the highest efficacy against bacteria in the contamination model, with a inactivation efficiency of 99.97%, whereas ultraviolet irradiation showed the highest efficacy against fungi in the contamination model, with a inactivation efficiency of 99.98%.

## Data Availability

The datasets presented in this study can be found in online repositories. The names of the repository/repositories and accession number(s) can be found at: https://www.ncbi.nlm.nih.gov/, PP783893-PP783915.
