# Supplementary material for: Study on disinfection using wet wipes and UV irradiation for areas prone to condensate accumulation in the Chinese Space Station
Source: Front Microbiol. 2026 Jun 16;17:1850068. doi: 10.3389/fmicb.2026.1850068 (PMC13314837; doi:10.3389/fmicb.2026.1850068)
Supplement: Supplementary file 1 [file Table_1.docx]

Supplementary Material

# Supplementary Figures and Tables


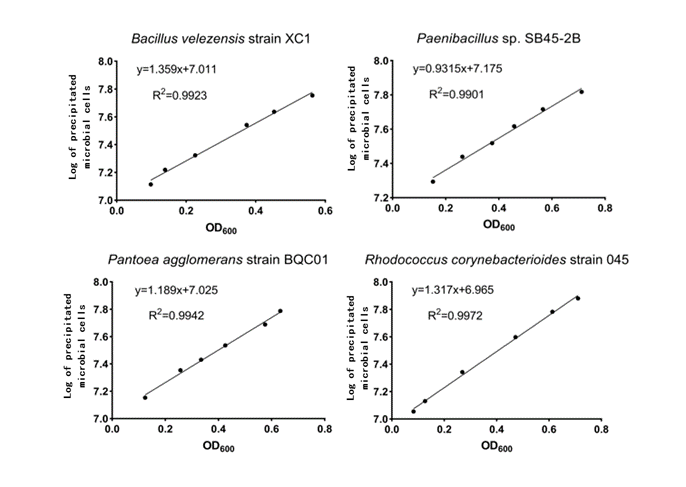


**Supplementary Figure 1.** Standard curve equations correlating cell abundance and OD_600_ for bacterial strains belonging to the genera Bacillus, Paenibacillus, Pantoea, and Rhodococcus. These four genera, which together accounted for the vast majority of the total bacterial population, were selected for construction of the standard curves. On this basis, we were able to establish a simulated contamination model representing microbial species from areas prone to microbial proliferation in the space station environment. The x-axis represents absorbance at 600 nm, and the y-axis represents the base-10 logarithm of cell abundance.

**Supplementary Table 1.** Residual Microbial Counts Obtained by Culture-Based Methods

|  | LB contact plate (CFU/100cm2) | PDA contact plate (CFU/100cm2) |
| --- | --- | --- |
| Control | 828.68(±39.092) | 155.56(±52.24) |
| Purewater | 657.32(±154.32) | 120.88(±58.00) |
| Alcohol | 170.24(±111.68) | 114.24(±57.44) |
| Ultraviolet radiation | 228.00(±67.08) | 63.12(±38.16) |
| Quaternary ammonium salt | 289.32(±65.00) | 122.68(±47.92) |

**Supplementary Table 2.** Microbial Residual Counts Determined by qPCR Method

|  | Bacterium qPCR (copy number/100cm2) | Fungi qPCR(copy number/100cm2) | Bacterium PMA-qPCR (copy number/100cm2) | Fungi PMA-qPCR(copy number/100cm2) |
| --- | --- | --- | --- | --- |
| Control | 2.92(±0.39)×10^8^ | 7.96(±0.28)×106 | 1.33(±0.04)×10^6^ | 9.91(±1.24)×10^4^ |
| Purewater | 1.27(±0.39)×10^8^ | 2.37(±0.23)×10^6^ | 9.29(±1.42)×10^5^ | 1.67(±0.11)×10^4^ |
| Alcohol | 2.96(±0.46)×10^7^ | 3.03(±0.48)×10^6^ | 8.25(±0.57)×10^3^ | 9.82(±0.44)×10^3^ |
| Ultraviolet radiation | 4.20(±0.36)×107 | 6.80(±0.32)×10^6^ | 7.21(±1.57)×10^4^ | 9.62(±0.56)×10^2^ |
| Quaternary ammonium salt | 6.00(±0.68)×107 | 3.19(±0.57)×10^6^ | 1.50(±0.13)×10^5^ | 5.10(±0.29)×10^3^ |
